# Empirical research on cognitive diagnosis of scientific argumentation ability based on the DINA model

**DOI:** 10.3389/fpsyg.2025.1701937

**Published:** 2026-01-21

**Authors:** Lou Baidan, Cui Yi

**Affiliations:** School of Teacher Education, Harbin Normal University, Harbin, China

**Keywords:** cognitive diagnosis, DINA model, educational measurement, empirical research, learning path, personalized learning, scientific argumentation

## Abstract

This study focuses on the cognitive diagnosis (CD) of scientific argumentation ability. A hierarchical model of six cognitive attributes was developed by expert cognitive analysis, utilizing the Toulmin Argument Model and SOLO Taxonomy theory. The research was conducted in two stages: In the first stage, a 33-item diagnostic test was developed based on Q-matrix theory, and the validity of the cognitive model and Q-matrix was verified using data from a sample of 240 tenth-grade students (M age = 15.6 years, SD = 0.72); and in the second stage, through multi-model fitting comparisons, the DINA model was identified as the optimal model, and students’ scientific argumentation ability was diagnosed. The diagnostic results demonstrated that the construction of the cognitive model and Q-matrix was valid and the DINA model could effectively diagnose the cognitive structure of scientific argumentation, further constructing students’ learning paths and providing empirical evidence for the targeted design of scientific argumentation thinking training. These outcomes transform general ability assessment into diagnosis of specific cognitive components, offering targeted evidence for instructional intervention. Overall, the study provides a reliable theoretical and empirical foundation for the assessment and improvement of scientific argumentation competency.

## 1 Introduction

Global science education is undergoing an in-depth transformation from a knowledge-based to a literacy-oriented approach. Cultivating citizens with scientific literacy has become a consensus direction in international science education reform. As a key component of scientific literacy, scientific argumentation refers to the process of citing evidence or data to support one’s own views or challenge others’ opinions, claims, or conclusions (Faize et al., 2017). The cultivation of students’ scientific concept ability aims to enable them not only to understand scientific knowledge but also to reason, question, defend, and revise viewpoints based on evidence such as scientists, thereby making rational decisions in the complex and ever-changing real world (Sampson and Blanchard, 2012). Therefore, scientific argumentation holds a fundamental position in several important science education frameworks and assessment systems: the Framework for K-12 Science Education (National Research Council, 2012) released by the National Research Council (NRC) in 2012 states that scientific argumentation is a cognitive process of constructing claims through chains of evidence and evaluating the effectiveness of scientific assertions, and a key bridge connecting scientific knowledge and practical application. In 2013, the Next Generation Science Standards (NGSS) (National Research Council, 2013) took “Science and engineering practices” as the primary dimension, requiring learners to develop the ability to construct scientific discourse systems and logical critical thinking through the complete argumentative structure of Claim-Evidence-Reasoning (CER). International assessments, such as PISA 2015 Scientific Literacy (Programme for International Student Assessment, 2015), TIMSS 2015 (Foy and Yin, 2015), and National Assessment of Educational Progress (NAEP) (National Assessment of Educational Progress (Project), National Institute of Education (US), 1983), by the Organization for Economic Co-operation and Development (OECD), have also incorporated scientific argumentation into their evaluation systems, reflecting the indispensability of scientific argumentation in cultivating the core scientific literacy of future citizens and its central position as the essence of scientific inquiry (Nussbaum, 2002).

Scientific argumentation is one of the most crucial skills for constructing a knowledge system. However, studies indicate that students worldwide generally lack scientific argumentation skills (Songsil et al., 2019), and such argumentation is seldom applied in the process of science learning (Kurniasari and Setyarsih, 2017; Muna and Rusmini, 2021; Rahayu et al., 2020). This issue is related to an imbalance between standards and assessment methods. Existing evaluation systems are primarily based on the Toulmin Argumentation Model (Toulmin, 2003), focusing on structural components such as “Claim–Evidence–Reasoning” to assess argumentation. Most of these methods emphasize judging the “structural completeness” of arguments (Chinn and Clark, 2013; Demircioglu et al., 2022; Backman et al., 2023; Noroozi et al., 2023; Zhai et al., 2023; Wilson et al., 2024), while overlooking the interactions among argumentative elements, differences in argumentative quality, and the developmental trajectory of students’ argumentation competence. Consequently, they fail to capture how students progressively construct and revise their viewpoints or how their argumentation strategies and thinking modes evolve—which is precisely the information most needed for instructional interventions. Therefore, how to deeply evaluate the quality and dynamic development of scientific argumentation thinking has become a key challenge in current research on scientific argumentation assessment.

The emergence of cognitive diagnosis (CD) offers a potential solution to this predicament. This theory transcends the limitations of static assessment in traditional testing methodologies. By analyzing test-taker performance on test items, it infers their mastery of specific cognitive attributes and further investigates the cognitive processes and structures employed by test-takers within specific domains (Rupp and Templin, 2008). Its core advantages manifest in three key aspects: first, through Q-matrix theory, it enables a more precise deconstruction of abilities into an observable cognitive model; second, it utilizes cognitive diagnosis model to accurately capture the knowledge structure developed by individuals throughout the learning process; and finally, it implements a generation mechanism for personalized diagnostic reports and learning path. This “deconstruction-diagnosis-intervention” framework directly addresses the requirements of scientific argumentation development for process-oriented diagnosis and targeted intervention, thereby offering theoretical feasibility for establishing dynamic and sophisticated evaluation systems.

In summary, cognitive diagnostic assessment of scientific argumentation holds significant research value. The primary objectives of this study are as follows: (a) examine the reasonableness of the proposed structural framework for scientific argumentation based on the analysis results of the cognitive diagnosis model; (b) demonstrate the practical applicability of this approach in identifying argumentation structures and proficiency levels through the analysis of middle school students’ argumentation performance; (c) and analyze the characteristics of students’ performance in scientific argumentation, to provide an actionable reference framework for assessing the quality of scientific argumentation.

## 2 Cognitive attributes and hierarchical relationships

Cognitive attributes and their hierarchical relationships form the core foundation of cognitive diagnosis. Cognitive attributes refer to the knowledge, skills, or strategies required for an individual to complete specific tasks or solve problems (Tatsuoka, 2009). In the process of individual cognitive processing, various cognitive attributes do not exist in isolation. Instead, they are interconnected based on the internal organizational structure of an individual’s knowledge representation or the logical structure among attributes, forming specific logical sequences or hierarchical relationships, which is referred to as attribute hierarchical relationship (Tu et al., 2019). Typically, by defining the cognitive attributes required for diagnostic objectives and constructing their hierarchical relationships through methods such as expert certification and literature research, researchers can more clearly reveal the internal logic of individual cognitive processing, thereby guiding the design and implementation of cognitive diagnosis tests (Wang et al., 2018). This study will categorize cognitive attributes and hierarchical relationships along two dimensions: the structure and level of scientific argumentation.

### 2.1 Analysis of the dimensional attributes of scientific argumentation structure

This study adopts Toulmin’s argumentation model as its analytical framework, primarily due to its dual strengths in scientific argumentation research and educational practice. This model not only possesses clear structural characteristics, enabling the decomposition of complex argumentation processes into manageable components, but more crucially, it emphasizes the refutability and conditional nature of scientific argumentation. This more accurately reflects the constructive essence of scientific knowledge—namely, that scientific claims advance through the support of evidence and warrant, followed by the application of backing, qualifier, and rebuttal (Brockriede and Ehninger, 1960). A claim represents the arguer’s stance or perspective on a specific scientific phenomenon or issue. Evidence serves as the basis for supporting the claim, comprising verifiable facts or data. The warrant articulates the logical link between evidence and claim, explaining how the evidence supports the claim. Backing offers general laws or principles that underpin the warrant, thereby supplementing and justifying its validity. The qualifier specifies the conditions or scope under which the claim is valid. A rebuttal identifies potential objections or counterarguments within the reasoning process, which may challenge the inference of the claim from the evidence and warrant (Jiménez-Aleixandre and Erduran, 2007). These elements exhibit a hierarchical structure within the argumentative framework: claim, evidence, and warrant are the fundamental and indispensable components of scientific argumentation; rebuttal represents the critical examination phase of the argument itself; and qualifier and backing are not independent argumentative elements but derivative ones that serve the core elements by clarifying the boundaries of claim and reinforcing warrant. Therefore, based on Toulmin’s argumentation model and integrating the practical application needs of cognitive diagnosis with the core elements of scientific argumentation, this study focuses on four core structural components: claim, evidence, warrant, and rebuttal, which are established as the basis for the cognitive attribute division of the scientific argumentation structure dimension in this study (Table 1).

**Table 1:** Scientific argumentation structure dimension cognitive attribute.

Number	Cognitive attribute	Connotation
A1	Claim formulation	Putting forward a clear and verifiable viewpoint or opinion based on phenomena or data.
A2	Evidence identification	Extracting specific facts that support a Claim from experimental data, observed phenomena, or literature.
A3	Warrant construction	Establishing a logical relationship between Evidence and Claim and explaining the reasoning process.
A4	Rebuttal formulation	Proposing and addressing potential counterexamples or objections.

### 2.2 Analysis of the dimensional attributes of scientific argumentation level

An analysis of existing scientific argumentation evaluation criteria (Corner and Hahn, 2009; Sampson and Clark, 2008) reveals that their level classification is primarily based on two key dimensions: (a) the scientific rigor of scientific argumentation content, namely the truthfulness and reliability of evidence, the rationality of warrant, and the rigor of the logical relationship between evidence, warrant, and claim; (b) the completeness of scientific argumentation structure elements, i.e., the number of Core elements (such as Evidence, Warrant, Rebuttal, etc.) included in the argument. Taking “Evidence” as an example, the more reasonable, reliable, and numerous the sources of Evidence provided by the arguer to support the claim, the stronger the support strength of their scientific argumentation usually is, and the credibility of the claim is correspondingly enhanced. These two dimensions, respectively, describe the “quality” and “quantity” of argumentation, but in practice they are often used independently, failing to fully reveal the integration and development of learners’ internal cognitive structures. Therefore, an integrated framework that can describe the complexity of cognitive structure is urgently needed.

The Structure of Observed Learning Outcomes (SOLO) taxonomy theory provides a framework for characterizing hierarchical levels of scientific argumentation from both “quality” and “quantity” perspectives (Biggs and Collis, 2014; Chan et al., 2002). This theory systematically categorizes the hierarchical levels of cognitive structures exhibited by individuals during learning tasks, emphasizing the intricate mechanisms of cognitive processing and the deep interconnections among its various elements, rather than merely making binary distinctions based on correctness or quantity. This theory classifies understanding levels into five hierarchical stages from low to high: pre-structural, unistructural, multistructural, relational structure, and extended abstract structure. These correspond, respectively, to the inability to understand the problem, understanding one aspect of the problem, understanding multiple aspects, establishing connections, and engaging in abstract thinking (Biggs and Collis, 1982). This description of cognitive structure complexity and integration degree aligns well with the requirements of scientific argumentation level for content rigor and structure completeness (Stålne et al., 2016). However, in practical cognitive diagnostic research, the “Pre-structural” level indicates that learners have not mastered any relevant cognitive skills or have made incorrect responses. This essentially constitutes a lack of cognitive attribute, which can be reflected by an extremely low attribute mastery probability. Therefore, it is not listed as an independent attribute to be diagnosed. Additionally, since both “Relational structure” and “Extended abstract structure” fall under higher-order thinking and are closely interrelated, they are merged into a single higher-order attribute. This not only better meets the practical needs of diagnosis of scientific argumentation ability but also simplifies the diagnostic model and enhances diagnostic accuracy. Therefore, based on the core ideas of the SOLO theory, this study has extracted and constructed three core cognitive attributes within the level dimension of scientific argumentation (Table 2).

**Table 2:** Cognitive attributes of the level dimension of scientific argumentation.

Number	Cognitive attribute	Connotation
B1	Unistructural Application	Put forward one relevant and logical Claim, Evidence, Warrant or Rebuttal
B2	Multistructural Application	Integrate multiple relevant and logical Claims, Evidences, Warrants or Rebuttals
B3	Associative Extended Structure	Relate argumentative relationships among multiple elements and synthesize arguments to draw valuable open conclusions or counterevidence

### 2.3 Construction of attribute hierarchical relationship in scientific argumentation

**Figure 1:**
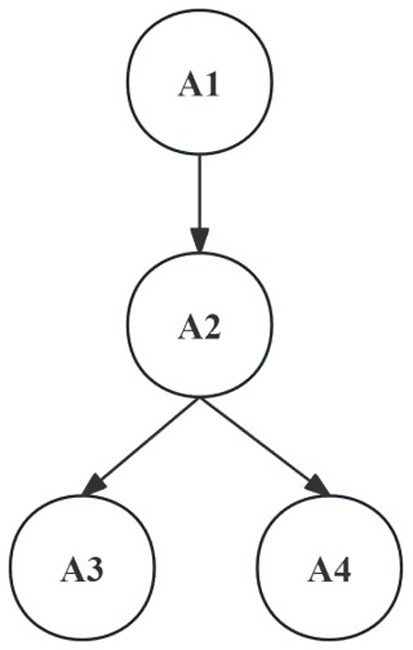
Hierarchical relationship of attributes in the structural dimension of scientific argumentation.

Based on expert discussions and validation, the hierarchical relationship of cognitive attributes of the structural dimension and the level dimension of scientific argumentation was constructed. Within the structural dimension of scientific argumentation, the arguer first needs to formulate a clear core view and accomplish the thinking transformation from observed phenomenon to construct hypothesis. Next, based on the claim, select supportive data, phenomena, or literature as evidence. Subsequently, strive to establish a logical connection between the evidence and the claim and articulate the scientific argumentation. Throughout the entire process, the arguer must conduct a critical review of the overall scientific argumentation process. Such scrutiny can not only be applied to the final stage of argumentation, critiquing the entirety of how the claim, evidence, and reasons are formed, but also involves questioning potential counterexamples or opposing evidence during the evidence screening phase. In summary, the structural dimension of scientific argumentation comprises four key components: claim formulation (A1), evidence identification (A2), warrant construction (A3), and rebuttal formulation (A4), which exhibit a specific attribute hierarchical relationship (Figure 1). Within the horizontal dimension of scientific argumentation, according to the SOLO Taxonomy theory, the arguer’s thinking progresses sequentially from single information point processing to multi-information point listing, and further to establishing logical association between information points while conducting abstract generalization or expansion. This manifests as a linearly progressive hierarchical structure, specifically B1 → B2 → B3.

**Figure 2:**
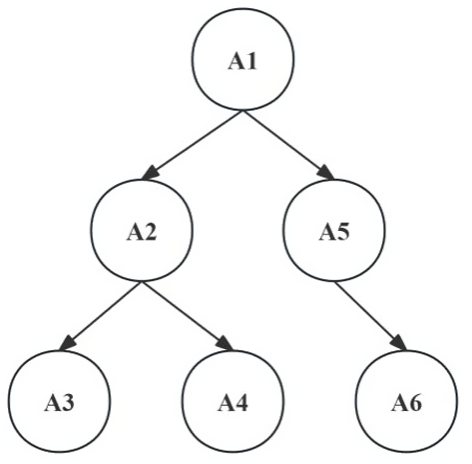
Hierarchical relationship of attributes of scientific argumentation.

Based on the above analysis and through discussions with educational experts, single-point structure application (B1) within the horizontal dimension focuses on students’ ability to apply a single scientific argumentation structure element. Consequently, this study integrates it with the four cognitive attributes of the structural dimension, resulting in four attributes: single claim proposal, single evidence identification, single reason construction, and single rebuttal proposal. Subsequently, by combining these with the cognitive attributes B2 and B3 of the horizontal dimension, a scientific argumentation cognitive diagnosis framework encompassing six core cognitive attributes is constructed. The specific classification of these attributes and their interrelationships is detailed in Table 3 and Figure 2.

**Table 3:** Scientific argumentation cognitive attribute.

Horizontal dimension	Structural dimension	Cognitive attribute	Connotation
B1	A1	Single Claim Formulation	Put forward a relevant and logical claim
	A2	Single Evidence Identification	Identify relevant and logical evidence
	A3	Single Warrant Construction	Construct a relevant and logical warrant
	A4	Single Rebuttal Formulation	Put forward a relevant and logical rebuttal
B2	A5	Multistructural Application	Integrate multiple relevant and logical claims, pieces of evidence, warrants or rebuttals
B3	A6	Associative Extended Structure	Establish connections between the argumentative relationships among multiple elements and synthesize arguments to draw valuable open conclusions or counterevidence.

## 3 Practice study 1: hierarchical relationships of attributes and Q-matrix authentication

### 3.1 Research purpose

This section mainly examines the scientific nature of cognitive attributes and their hierarchical relationships through small-scale oral reporting and paper-and-pencil quizzes, constructs, and authenticates the effectiveness of Q-matrix, ensuring that cognitive diagnosis outcomes can accurately depict individuals’ cognitive presence information and realize the transformation from theory to practice.

### 3.2 Methodology and sample selection

First, based on the determined hierarchical structure of cognitive attributes, analyze the direct relationships between attributes to form the adjacency matrix (A), and then, through Boolean computation, calculate the indirect and self-relationships of attributes to obtain the reachable matrix (R). Based on the reachable matrix, the augmentation algorithm was adopted to determine the ideal measurement pattern (Cai and Tu, 2015), based on which the Q-matrix was constructed (Tatsuoka, 1983), and further developed into the cognitive diagnostic test paper for scientific argumentation ability. Subsequently, 240 first-grade students from two senior high schools in Harbin were selected to take the test. At this phase, students had already acquired a certain foundation of scientific knowledge and could understand the test content. Meanwhile, six students were selected to conduct oral reporting, and their thinking during the answering process was recorded through the think-aloud protocol to conduct an in-depth investigation of the cognitive process. Finally, students’ response data were collected, and the SPSS software was used to analyze the difficulty, discrimination, and reliability of analytics artifacts, and compute the Hierarchical Consistency Index (HCI) to assess the matchmaking degree between the actual item response pattern (IRP) and the ideal response pattern.

### 3.3 Measurement tools

The measurement tool is a cognitive diagnostic test paper developed based on the Q-matrix. The Q-matrix was designed to ensure that each cognitive attribute was assessed by at least three items. To control for measurement error, three experts in science education independently reviewed the association between each item and its corresponding attributes, reaching unanimous agreement. This process indicates strong content validity of the Q-matrix, ultimately forming the scientific argumentation Q matrix (33 questions × 6 attributes, Table 4). In the Q-matrix, if question N measures attribute K, it is recorded as QNK = 1; if not measured, it is recorded as QNK = 0. For example, initiatives Item2-3, Item6-3, and Item10-3 (110011) measure A1, A2, A5, and A6; while initiatives Item3-1, Item7-1, and Item11-1 (111000) measure A1, A2, and A3.

**Table 4:** Q matrix.

Item	A1	A2	A3	A4	A5	A6
Item1-1	1	0	0	0	0	0
Item1-2	1	0	0	0	1	0
Item2-1	1	1	0	0	0	0
Item2-2	1	1	0	0	1	0
Item2-3	1	1	0	0	1	1
Item3-1	1	1	1	0	0	0
Item3-2	1	1	1	0	1	0
Item3-3	1	1	1	0	1	1
Item4-1	1	1	1	1	0	0
Item4-2	1	1	1	1	1	0
Item4-3	1	1	1	1	1	1
Item5-1	1	0	0	0	0	0
Item5-2	1	0	0	0	1	0
Item6-1	1	1	0	0	0	0
Item6-2	1	1	0	0	1	0
Item6-3	1	1	0	0	1	1
Item7-1	1	1	1	0	0	0
Item7-2	1	1	1	0	1	0
Item7-3	1	1	1	0	1	1
Item8-1	1	1	1	1	0	0
Item8-2	1	1	1	1	1	0
Item8-3	1	1	1	1	1	1
Item9-1	1	0	0	0	0	0
Item9-2	1	0	0	0	1	0
Item10-1	1	1	0	0	0	0
Item10-2	1	1	0	0	1	0
Item10-3	1	1	0	0	1	1
Item11-1	1	1	1	0	0	0
Item11-2	1	1	1	0	1	0
Item11-3	1	1	1	0	1	1
Item12-1	1	1	1	1	0	0
Item12-2	1	1	1	1	1	0
Item12-3	1	1	1	1	1	1

This study referred to existing scientific argumentation test questions (Han, 2016; Sampson and Schleigh, 2013; Frey et al., 2015), and conducted targeted adaptation based on the Q-matrix associated with cognitive attribute, ultimately forming a cognitive diagnostic test paper containing three scientific argumentation contexts with a total of 12 questions. An example of the question is as follows:

On 7 October 2013, three scientists, Marc Van Montagu, Mary-Dell Chilton, and Robert Fraley, received the World Food Prize for their work on genetically modified organisms (GMOs). The prize honored their work on technologies that improve food productivity and availability while addressing difficulties posed by global population increase and climate change. Meanwhile, scientists in the Philippines were developing rice types to address one of the world’s most serious health problems: vitamin A deficiency. Because of its color, this biofortified rice was dubbed “Golden Rice.” The granting of the prize to the three scientists, however, drew criticism from those who question the safety and benefit of genetically modified organisms. Anti-GMO sentiment grew stronger in places such as the Philippines, where demonstrators damaged experimental plots of genetically altered rice intended to treat vitamin A deficiency. Opposition groups, notably Greenpeace, strongly opposed the research. Proponents of transgenic rice claim that the crop has the potential to save up to 2 million lives every year. Patrick Moore, an ecologist and former Greenpeace president, supports its development, arguing that the technology should not be disputed while children continue to die from starvation. Greenpeace, on the other hand, contends that the majority of genetically modified crops sold in developing nations are herbicide- or insect-resistant, and claims that this agricultural paradigm endangers human health, farmer livelihoods, and the environment.

1. Based on the data in the above graphs, what conclusions can you draw?
2. Please select one of the conclusions you obtained in (1) and write down all the evidence that supports this conclusion.
3. Please describe how the evidence in (2) supports the corresponding conclusion.
4. If someone wants to rebut your conclusion and evidence in (2), please conjecture and write down what his counter-argument, along with its possible evidence and warrant, might be.

### 3.4 Result analysis

Analysis of oral reports: After sorting out and analyzing the oral reports of six students, it was found that students generally went through four stages in the cognitive process of the scientific argumentation structure dimension: first, reading materials to extract the core claim; second, identifying the relevant evidence in the materials; third, using existing knowledge to provide supporting warrant for the argument; finally, attempting to conduct reverse thinking and perform rebuttal on their own viewpoints. The analysis revealed that two students had difficulty clearly distinguishing between evidence and supporting warrants in their oral expressions, but they could basically distinguish them after sorting out the requirements of the topic. This indicates that the present cognitive attributes of scientific argumentation structure cognitive attributes, and its hierarchical relationship division are reasonable. In the scientific argumentation level dimension, five students could stably reach the B1 level, two students could stably reach the B2 level, and the B3 level was almost not achieved. Considering that the current sample size of oral reports is small and the B3 level has high requirements for scientific argumentation ability, the low achievement rate is a reasonable phenomenon. In conclusion, the scientific argumentation, cognitive attributes, and their hierarchical relationship set in this study are reasonable.

Paper-and-pencil test analysis: The paper-and-pencil test response data of 240 students were collected, and a 0–1 response matrix with 240 columns (students) × 33 rows (items) was constructed (marked as 1 for correct answers and 0 for incorrect answers). All assessments were scored according to detailed rubrics based on the Q-matrix and predefined cognitive attributes. The scoring was performed independently by a researcher who had undergone systematic training. Through repeated calibration, the rater thoroughly mastered the scoring criteria. To evaluate the reliability of the scoring, a partial sample retest method was employed. The same rater independently rescored a randomly selected 20% of the samples to assess intra-rater reliability. The results indicated good consistency (Kappa = 0.85, *p* < 0.01). The test difficulty analysis showed that the overall test paper difficulty value was 0.4141, which was at a moderate level. Among them, 52% of the items had a difficulty between 0.2 and 0.8, 18% of the items were relatively easy (difficulty > 0.8), and 30% of the items were relatively difficult (difficulty < 0.2). Further analysis revealed that the number of scientific argumentation cognitive attributes examined by the items was positively correlated with their difficulty. That is, the more attributes an item examines, the higher the item’s difficulty. This result may be related to students’ proficiency in the comprehensive application of multiple attributes in scientific argumentation. The overall discrimination coefficient was 0.5480, among which 97% of question discrimination was greater than 0.4, indicating that the test paper could well distinguish students of different ability levels. The Cronbach’s alpha was 0.939, indicating excellent reliability. For the attribute hierarchy consistency, the HCI proposed by Cui and Leighton (2009) was used to test the rationality of the attribute hierarchy. Wang and Gierl (2007) pointed out that an average HCI value greater than 0.6 indicates a reasonable hierarchy division. The average value of HCI calculated in this test is 0.7098, which supports the rationality of the attribute hierarchical relationship.

Combining the results of the oral report and paper-and-pencil test analysis, the scientific argumentation cognitive attributes system and its hierarchical relationship set in this study were verified, indicating that it is reasonable. Based on this, subsequent model selection for cognition diagnosis analysis can be conducted.

## 4 Practical study 2: cognitive diagnostic research on scientific argumentation ability

### 4.1 Research purpose

This study aims to select an appropriate cognitive diagnosis model to assess the scientific argumentation ability of the subjects, and demonstrate the feasibility of using cognitive diagnostics to evaluate scientific argumentation skills.

### 4.2 Methodology

This study aims to evaluate the scientific argumentation ability of the subjects through cognitive diagnosis. The core task is to select a suitable cognitive diagnosis model to complete this assessment. The research adopts the validation paradigm proposed by Chen and Thissen (1997) to construct a testing framework. The specific process is as follows:

First, model fit tests are used to screen the cognitive diagnosis model suitable for the test data. In view of the multidimensionality of scientific argumentation ability and the complexity of interactions among latent attributes, this study does not pre-specify a single model, but systematically compares representative models with different assumptions (such as compensatory and non-compensatory), such as G-DINA, DINA, and DINO. Second, conduct item fit tests and person fit tests on the initially screened candidate models to further verify their fit with the data, and finally determine the optimal model. After selecting the optimal model, use this model to estimate the attribute mastery probability of each subject. Based on the estimation results, classify the subjects into corresponding attribute mastery patterns. Finally, by analyzing the distribution and traits of different attribute mastery patterns, reveal the scientific argumentation ability structure of the subject group and construct the learning path of students’ scientific argumentation.

### 4.3 Model selection

This study used the FlexCDMs platform (Tu et al., 2023) for the initial screening of the cognitive diagnostic model (CDM). The final model selection was based on a comprehensive evaluation of multiple fit indices, including test-level, item-level, and person-level fit statistics. Specifically, at the test fit test, model fit was assessed using four criteria: Akaike information criterion (AIC), Bayesian information criterion (BIC), Number of parameters (Npars), and Deviance. Among them, AIC and BIC achieve optimal selection by balancing model goodness-of-fit and complexity, and are the core indicators for model selection; Npars reflects model complexity, and Deviance reflects the degree of deviation between the model and the data, which can be used as auxiliary indicators. All four indicators follow the principle that “the smaller the value, the better the fit,” based on the comparison results (Table 5).

**Table 5:** Model fitting data.

Model	Npars	Deviance	AIC	BIC
ACDM	213	5179.23	5605.23	6346.61
DINA	129	5478.84	5736.84	6185.84
DINO	129	5951.35	6209.35	6658.35
GDINA	669	4653.1	5991.1	8319.65
GDM	172	5209.4967	5553.4967	6152.1666
LCDM	628	4938.5123	6194.5123	8380.3536
LLM	213	4886.4	5312.4	6053.78
RRUM	213	4953.65	5379.65	6121.02

The item fit test is a key step to measure the fit between the model and each item in the test paper, which is directly related to the accuracy of diagnostic results. This study used root mean square error of approximation (RMSEA) as the core indicator, and according to the standardized evaluation criteria established by Oliveri and Von Davier (2011), the RMSEA critical value was set to 0.1; that is, when the RMSEA of an item is ≤ 0.1, it indicates that the model can effectively restore the response pattern of that item. Based on the data presented in Table 5, the model demonstrating superior fit was selected for further examination. When the DINA model was applied, the obtained RMSEA was 0.076, which demonstrated the best fit among all models, indicating good overall item-level model fit. Subsequently, person fit was evaluated, typically using the lz statistic. A response pattern is flagged as misfitting when lz < −2. In this study, under the DINA model, there were 14 subjects with lz < −2 in the sample of this study, meaning that 94% of the subjects’ response patterns were consistent with the expectations of the DINA model, demonstrating a relatively better fit among all the models.

Comprehensive results of the model’s relative goodness of fit, item fit, and person fit show that the DINA model (Junker and Sijtsma, 2001) exhibited superior adaptability. This model can effectively characterize the cognitive traits of test items and the response patterns of subjects. Of particular importance is the inherent “non-compensatory” trait of the model—that is, mastering all necessary cognitive attributes is a prerequisite for correct answers, which is highly consistent with the essence of scientific argumentation ability that emphasizes the integrity of the logical chain and the indispensability of each link, and helps to accurately locate students’ defects in the scientific argumentation process (Cai et al., 2013; De La Torre, 2011). Therefore, this study ultimately adopted the DINA model to carry out the excess syndrome/pattern analysis of cognitive diagnosis for the problem-solving ability of experimental inquiry questions.

### 4.4 Result analysis

Attribute mastery probability: The DINA model was used to estimate students’ mastery probability of each attribute, as shown in Table 6. Overall, attribute mastery probabilities are reasonably distributed and align with the theoretical hierarchy of scientific reasoning abilities. This indicates that the DINA model can effectively identify and differentiate mastery levels of cognitive attributes with varying complexities, demonstrating scientific rigor and applicability in multidimensional competency assessment. Specific mastery probability analysis is as follows: single evidence identification (A2,92.52%) and single claim formulation (A1,92.50%) had the highest mastery probabilities, forming the first level. In contrast to single claim formulation (A1), single evidence identification (A2) was found to have significantly higher mastery rates (95.52% vs. 92.50%). A potential explanation may be that high school science classes often train students to extract direct evidence from data or texts (A2) more frequently and explicitly, whereas there seems to be a relative lack of systematic training in independently proposing claims (A1), which requires creative thinking and expressive confidence. The mastery rates of single warrant construction (A3,50.14%) and single rebuttal formulation (A4,55.73%) are relatively lower mastery rates compared to basic skills, reflecting that students are relatively weak in the logical chain connecting claims and evidence (A3) and the ability of critical questioning (A4). This is consistent with the characteristic that high school students’ formal operational thinking is still developing, and constructing rigorous reasoning and actively challenging presuppositions requires more targeted training. The mastery probability of multistructural application (A5) is 47.09%, revealing students’ difficulties in information organization and structured expression; the mastery probability of associative extended structure (A6) is the lowest at 36.43%, which may indicate challenges in systematic analysis, transfer application, and generating innovative conclusions. These lower mastery rates could be related to the general scarcity of practical opportunities for cross-element association and open-ended conclusion derivation in typical high school instruction, which often focuses more on knowledge transmission and standardized answers, and may also be limited by the developmental stage of students’ abstract thinking and complex problem-solving abilities. Taken together, the findings provisionally suggest that current teaching appears relatively effective in training basic skills such as evidence identification, while potential deficiencies are observed in warrant construction, critical rebuttal, and the more advanced B2 level and B3 level competencies, areas that may urgently need strengthening.

**Table 6:** Attribute mastery probability.

B1				B2	B3
A1	A2	A3	A4	A5	A6
0.9250	0.9252	0.5014	0.5573	0.4709	0.3643

Attribute mastery pattern: In this study, the DINA model and the maximum likelihood estimation (MLE) method were used to diagnose the subjects’ attribute mastery pattern. This method performs parameter estimation by maximizing the likelihood function of observed data, without the need to preset the attribute prior distribution, and is computationally efficient. The ideal mastery pattern of cognitive diagnosis was obtained through the augmentation algorithm (Table 7). Analysis of the diagnosis results shows that 67% of students’ knowledge state belonged to the ideal mastery pattern. Table 7 details the number distribution of various mastery patterns, among which the non-ideal mastery pattern is marked with “*.” According to the data in the table, students’ scientific argumentation ability shows significant structural and level differentiation. Among them, 53 subjects mastered all attributes simultaneously (pattern 111,111), accounting for the highest proportion, indicating that these students can effectively coordinate the structural elements of scientific argumentation and carry out multi-level integrated application. A large number of subjects mastered the scientific argumentation structure but did not reach the level of association extension (e.g., pattern 111,110, 24 subjects), reflecting that the training of single scientific argumentation skill in senior high school teaching is relatively solid, but there is still a significant deficiency in guiding students to carry out multi-element association and open-ended conclusion generation ability culture. It is worth paying close attention to some non-ideal mastery patterns. For example, 110,001 (19 subjects) and 110,101 (23 subjects) mastered the attribute of A6 without mastering A5. This cognition structure loss of balance may stem from guessing bias or question context hints in subjective question types, where students can piece together answers without a solid foundation. Overall, the distribution of attribute mastery patterns reflects both typical pathways in argumentation skill development and unconventional cognitive structures. This demonstrates the DINA model’s effectiveness and validity in precisely characterizing individual cognitive traits.

**Table 7:** Attribute mastery pattern and number of subjects.

Mastery pattern	Number of subjects	Mastery pattern	Number of people	Mastery pattern	Number of subjects	Mastery pattern	Number of people
000000	0	000001*	2	111,010	8	010101*	1
100,000	1	000010*	1	111,011	7	011011*	1
110,000	12	000101*	1	110,110	6	011100*	3
111,000	10	000110*	1	110,111	7	011101*	1
110,100	12	000111*	1	111,110	24	101000*	1
100,010	0	001100*	2	111,111	53	110001*	19
100,011	0	001101*	1			110101*	23
110,010	6	001110*	1			111001*	8
110,011	6	010011*	1			111101*	10
111,100	9	010100*	1				

“*” indicates a non-ideal mastery mode.

**Figure 3:**
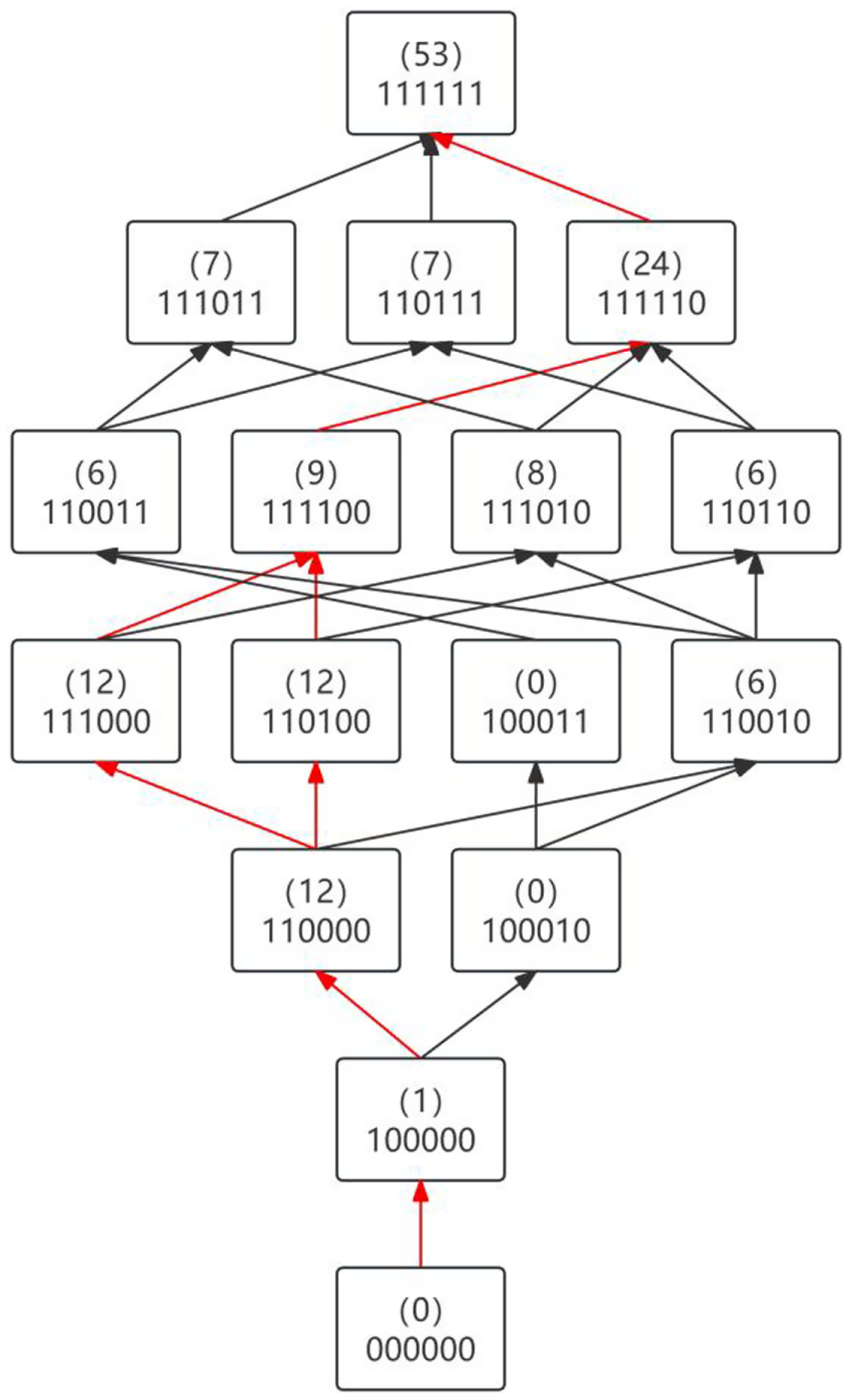
Learning path of scientific argumentation.

Learning path: Classified according to the hierarchical relationship of the number of mastered attributes in the knowledge state. For example, the state of not mastering any attribute (000000) is level 0, the state of mastering only 1 attribute (100000) is level 1, and so on, eventually forming a total of seven levels from level 0 to level 6. Table 8 integrates students’ knowledge state, corresponding attribute mastery level, and sample size distribution. To clearly depict the cognitive development sequence, path construction follows two principles: only considering first-order path between adjacent levels to avoid multi-level transition caused by unclear mastery order; and only considering path relationships among the 16 ideal mastery patterns (i.e., non-ideal states caused by none guessing and none forgetting) and excluding adverse paths where mastery (1) reverses to non-mastery (0). Based on the cognitive attribute hierarchical relationship, a learning path diagram was drawn (Figure 3). In the diagram, the second row of the rectangular box indicates the cognitive state, and the number in parentheses in the first row represents the sample size of that state. The path with the largest sample size is highlighted in red.

**Table 8:** Knowledge state, attribute mastery level and number of subjects.

Knowledge state	Attribute mastery level	Number of people	Knowledge state	Attribute mastery level	Number of people
000000	0	0	110,011	4	6
100,000	1	1	111,100	4	9
110,000	2	12	111,010	4	8
111,000	3	10	111,011	5	7
110,100	3	12	110,110	4	6
100,010	2	0	110,111	5	7
100,011	3	0	111,110	5	24
110,010	3	6	111,111	6	53

Analysis revealed that the two red-highlighted learning paths covered a total of 111 samples. The majority of students (69%) exhibited a mastery pattern consistent with a typical developmental sequence, which was the most common pattern observed and is thus hypothesized as a potential trunk path. For the development of students’ scientific argumentation ability. Based on the inferred mastery order observed in this cohort, a potential developmental sequence for scientific argumentation ability can be hypothesized: students first develop the basic abilities of single claim formulation (A1) and single evidence identification (A2); then transition to the ability of single warrant construction (A3) or single rebuttal formulation (A4); afterwards, they complementarily develop the ability of single rebuttal formulation (A4) or single warrant construction (A3); and finally point to the higher-order multistructural application (A5) and associative extended structure (A6). This hypothesized sequence aligns with the internal logic of scientific argumentation, which is also consistent with a pattern of gradual cognitive development. However, the analysis shows that some students did not follow the trunk path. Their mastery patterns appear to present two types of characteristics: first, apparent jumps in mastery, manifested as knowledge states skipping adjacent levels (e.g., moving directly from mastering only A1 to mastering A4), which may result from agile thinking or intuitive leaps based on prior knowledge; and second, unconventional combinations of attribute mastery, such as mastering higher-order attributes without having mastered basic ones, which could reflect conceptual misunderstandings or fragmented knowledge structures rather than representative developmental progress. Given the cross-sectional design, these interpretations remain provisional. For students with leapfrog development, it is recommended to adopt dynamic assessment to continuously track their cognitive trajectory and provide advanced challenge tasks that are adapted to their characteristics of rapid thinking. For students with unconventional attribute combinations, it is necessary to detect their potential confusion points or wrong concepts through an in-depth cognitive diagnosis interview or targeted test, and implement precise intervention to bridge the gap in basic cognitive structure. These findings further demonstrate that the DINA model not only depicts conventional developmental trajectories but also captures the uniqueness of individual cognitive development, providing empirical support for differentiated instructional interventions.

## 5 Limitations and future work

This study conducted cognitive diagnosis on scientific argumentation ability based on the DINA model and achieved certain results, but there are still several limitations that should be noted: (a) Although the 0–1 scoring mechanism adopted by the model simplifies the analysis process, the binary division may make it difficult to accurately depict the continuous development trait of scientific argumentation ability. In the future, the multi-level scoring model can be introduced to distinguish the depth of attribute mastery, so as to distinguish the depth levels of attribute mastery. For example, refutation ability can be subdivided into different levels, such as identify counter-examples and construct defenses. (b) The test sample was only taken from students of two high schools in Harbin, and the singularity of regional and curriculum backgrounds, as well as the sample size, may affect the generalizability of the conclusions (Zhan et al., 2016). Some students’ exposure to extracurricular “Non - curriculum standard content” was not taken as a control variable, which may interfere with the accuracy of attribute mastery probability estimation. In future, it is necessary to expand the sample size to a wider geographical area and diverse teaching backgrounds. Test material design also needs to balance multiple scientific contexts to enhance the representativeness of the results. (c) Subjective question types have limitations in measuring scientific argumentation ability, and question hints or guessing strategies may lead to misjudgment, resulting in students showing mastery of high-level abilities without mastering basic scientific argumentation elements. In future, examination forms can be increased, and the rigor and innovation of argumentative texts can be enhanced to more truly reflect the level of integration ability. (d) Although the learning path derived from the research conforms to the development law of most students, it fails to capture the dynamic process of individual cognitive leaps. For some students who exhibit the attribute mastery jump phenomenon or the abnormal combination pattern, it is necessary to clarify whether they belong to cognitive advantage or conceptual misunderstanding through longitudinal tracking. (e) The practical connection of the transformation of diagnostic results into teaching is still insufficient. The identified weak points in scientific argumentation urgently require targeted intervention. Personalized tasks can be pushed based on students’ cognition status, and a control experiment can be designed to compare the effects of conventional teaching, forming a “Diagnosis - path - intervention” closed loop. (f) At the same time, attention should be paid to the impact of teacher attitude on students’ scientific argumentation. Studies have shown that teachers’ own learning goals have a significant impact on scientific argumentation teaching, which may lead to differentiation in the development of students’ scientific argumentation ability (Knight-Bardsley and McNeill, 2016; Cinici, 2016). These in-depth issues require the reconstruction of a cognitive model from an educational perspective, so that the diagnostic system not only conforms to the essence of scientific argumentation but also responds to the complexity of real teaching scenarios.

## 6 Conclusion

This study systematically validated the scientific rigor and applicability of the diagnostic model through theoretical construction, statistical testing, and result interpretation.

Theoretically, this study employs the Toulmin argumentation model as its structural framework. Integrating SOLO taxonomy theory’s description of cognitive levels, it delineates the developmental trajectory of scientific argumentation ability—transitioning from a single-point structure to an interconnected, expansive structure. This design ensures the completeness of argumentative elements while reflecting the hierarchical nature of cognitive development, providing a robust theoretical foundation for defining attributes.

Statistically, multiple measurement indicators support the validity of the cognitive diagnosis. The Hierarchical Consistency Index (HCI) meets measurement requirements, and multiple fit indices indicate good model fit, demonstrating that the Q matrix effectively represents the cognitive demands of scientific argumentation. Furthermore, the DINA model was selected for cognitive diagnosis based on its fit performance. This model emphasizes the mechanisms of item response consistency and integrity, aligning closely with the inherent requirements of scientific argumentation for logic and evidence. This ensures the substantive validity of diagnostic results at the measurement model level.

Regarding result interpretation, diagnostic outcomes not only reveal students’ characteristics in scientific reasoning abilities, but their interpretability and value for intervention guidance further validate the applicability of cognitive diagnostics. Compared to traditional assessment methods that focus solely on overall performance or single scores, cognitive diagnostics can reveal the cognitive components underlying students’ external performance through attribute mastery patterns. This transforms general ability evaluations into diagnoses of mastery of specific cognitive elements, providing more targeted grounds for instructional intervention. Diagnostic results indicate higher mastery probabilities for argumentation structure elements (A1–A4), likely because these involve concrete mental operations that can be enhanced through repeated practice. Conversely, attributes requiring deeper cognitive processing, such as B2 and B3, exhibit lower mastery probabilities. This discrepancy not only aligns with fundamental cognitive development principles but, more importantly, vividly illustrates both strengths and weaknesses in students’ scientific argumentation abilities at the micro-cognitive component level. The distribution of attribute mastery patterns further reveals both individual differences and common patterns in students’ cognitive structures. Most students follow a progressive path from simpler to more complex attributes, confirming the typical stepwise development of scientific reasoning abilities. Simultaneously, the model identifies a minority of non-conventional mastery pattern—such as students demonstrating adequate grasp of complex attributes while failing to master certain foundational ones. These anomalous patterns may indicate unique thinking strategies or underlying misconceptions, highlighting the diagnostic model’s precision and validity in characterizing individual cognitive traits. Furthermore, the two primary learning pathways derived from the diagnostic results not only align with the developmental progression from lower to higher levels but also correspond to the anticipated hierarchical structure of scientific reasoning abilities. This indicates that the diagnostic findings possess clear cognitive developmental implications, providing empirical support for tiered instruction and personalized interventions.

In summary, this study systematically demonstrates the validity and scientific rigor of the constructed cognitive diagnostic model for assessing scientific argumentation abilities across multiple dimensions. The diagnostic findings not only reveal the cognitive structures underlying students’ observable behaviors but also provide reliable theoretical frameworks and practical pathways for refining the assessment, diagnosis, and instructional improvement of scientific argumentation competencies.

## Data Availability

The original contributions presented in the study are included in the article/Supplementary material, further inquiries can be directed to the corresponding author/s.
