# Supplementary material for: Empirical research on cognitive diagnosis of scientific argumentation ability based on the DINA model
Source: Front Psychol. 2026 Jan 21;16:1701937. doi: 10.3389/fpsyg.2025.1701937 (PMC12868137; doi:10.3389/fpsyg.2025.1701937)
Supplement: Supplementary file 3 [file Table_3.DOCX]

| **Number** | **Cognitive attribute** | **Connotation** |
| --- | --- | --- |
| A1 | Claim Formulation | Putting forward a clear and verifiable viewpoint or opinion based on phenomena or data. |
| A2 | Evidence Identification | Extracting specific facts that support a Claim from experimental data, observed phenomena, or literature. |
| A3 | Warrant Construction | Establishing a logical relationship between Evidence and Claim and explaining the reasoning process. |
| A4 | Rebuttal Formulation | Proposing and addressing potential counterexamples or objections. |

Table 1 Scientific argumentation structure dimensionCognitive attribute

Table 2 Cognitive Attributes of the Level Dimension of Scientific argumentation

| **Number** | **Cognitive attribute** | **Connotation** |
| --- | --- | --- |
| B1 | Unistructural Application | Put forward one relevant and logical Claim, Evidence, Warrant or Rebuttal |
| B2 | Multistructural Application | Integrate multiple relevant and logical Claims, Evidences, Warrants or Rebuttals |
| B3 | Associative Extended Structure | Relate argumentative relationships among multiple elements and synthesize arguments to draw valuable open conclusions or counterevidence |

Table 3 Scientific argumentationCognitive attribute

| **Horizontal dimension** | **Structural dimension** | **Cognitive attribute** | **Connotation** |
| --- | --- | --- | --- |
| B1 | A1 | Single Claim Formulation | Put forward a relevant and logical claim |
|  | A2 | Single Evidence Identification | Identify relevant and logical evidence |
|  | A3 | Single Warrant Construction | Construct a relevant and logical warrant |
|  | A4 | Single Rebuttal Formulation | Put forward a relevant and logical rebuttal |
| B2 | A5 | Multistructural Application | Integrate multiple relevant and logical claims, pieces of evidence, warrants or rebuttals |
| B3 | A6 | Associative Extended Structure | Establish connections between the argumentative relationships among multiple elements and synthesize arguments to draw valuable open conclusions or counterevidence. |

Table 4 Q Matrix

|  | **A1** | **A2** | **A3** | **A4** | **A5** | **A6** |
| --- | --- | --- | --- | --- | --- | --- |
| Item1-1 | 1 | 0 | 0 | 0 | 0 | 0 |
| Item1-2 | 1 | 0 | 0 | 0 | 1 | 0 |
| Item2-1 | 1 | 1 | 0 | 0 | 0 | 0 |
| Item2-2 | 1 | 1 | 0 | 0 | 1 | 0 |
| Item2-3 | 1 | 1 | 0 | 0 | 1 | 1 |
| Item3-1 | 1 | 1 | 1 | 0 | 0 | 0 |
| Item3-2 | 1 | 1 | 1 | 0 | 1 | 0 |
| Item3-3 | 1 | 1 | 1 | 0 | 1 | 1 |
| Item4-1 | 1 | 1 | 1 | 1 | 0 | 0 |
| Item4-2 | 1 | 1 | 1 | 1 | 1 | 0 |
| Item4-3 | 1 | 1 | 1 | 1 | 1 | 1 |
| Item5-1 | 1 | 0 | 0 | 0 | 0 | 0 |
| Item5-2 | 1 | 0 | 0 | 0 | 1 | 0 |
| Item6-1 | 1 | 1 | 0 | 0 | 0 | 0 |
| Item6-2 | 1 | 1 | 0 | 0 | 1 | 0 |
| Item6-3 | 1 | 1 | 0 | 0 | 1 | 1 |
| Item7-1 | 1 | 1 | 1 | 0 | 0 | 0 |
| Item7-2 | 1 | 1 | 1 | 0 | 1 | 0 |
| Item7-3 | 1 | 1 | 1 | 0 | 1 | 1 |
| Item8-1 | 1 | 1 | 1 | 1 | 0 | 0 |
| Item8-2 | 1 | 1 | 1 | 1 | 1 | 0 |
| Item8-3 | 1 | 1 | 1 | 1 | 1 | 1 |
| Item9-1 | 1 | 0 | 0 | 0 | 0 | 0 |
| Item9-2 | 1 | 0 | 0 | 0 | 1 | 0 |
| Item10-1 | 1 | 1 | 0 | 0 | 0 | 0 |
| Item10-2 | 1 | 1 | 0 | 0 | 1 | 0 |
| Item10-3 | 1 | 1 | 0 | 0 | 1 | 1 |
| Item11-1 | 1 | 1 | 1 | 0 | 0 | 0 |
| Item11-2 | 1 | 1 | 1 | 0 | 1 | 0 |
| Item11-3 | 1 | 1 | 1 | 0 | 1 | 1 |
| Item12-1 | 1 | 1 | 1 | 1 | 0 | 0 |
| Item12-2 | 1 | 1 | 1 | 1 | 1 | 0 |
| Item12-3 | 1 | 1 | 1 | 1 | 1 | 1 |

Table 5 Model Fitting Data

|  | **Npars** | **Deviance** | **AIC** | **BIC** |
| --- | --- | --- | --- | --- |
| ACDM | 213 | 5179.23 | 5605.23 | 6346.61 |
| DINA | 129 | 5478.84 | 5736.84 | 6185.84 |
| DINO | 129 | 5951.35 | 6209.35 | 6658.35 |
| GDINA | 669 | 4653.1 | 5991.1 | 8319.65 |
| GDM | 172 | 5209.4967 | 5553.4967 | 6152.1666 |
| LCDM | 628 | 4938.5123 | 6194.5123 | 8380.3536 |
| LLM | 213 | 4886.4 | 5312.4 | 6053.78 |
| RRUM | 213 | 4953.65 | 5379.65 | 6121.02 |

Table 6 Attribute mastery probability

| **B1** | | | | **B2** | **B3** |
| --- | --- | --- | --- | --- | --- |
| **A1** | **A2** | **A3** | **A4** | **A5** | **A6** |
| 0.9250 | 0.9252 | 0.5014 | 0.5573 | 0.4709 | 0.3643 |

Table 7 Attribute mastery pattern and Number of Subjects

| **Mastery Pattern** | **Number of Subjects** | **Mastery Pattern** | **Number of People** | **Mastery Pattern** | **Number of Subjects** | **Mastery Pattern** | **Number of People** |
| --- | --- | --- | --- | --- | --- | --- | --- |
| 000000 | 0 | 000001* | 2 | 111010 | 8 | 010101* | 1 |
| 100000 | 1 | 000010* | 1 | 111011 | 7 | 011011* | 1 |
| 110000 | 12 | 000101* | 1 | 110110 | 6 | 011100* | 3 |
| 111000 | 10 | 000110* | 1 | 110111 | 7 | 011101* | 1 |
| 110100 | 12 | 000111* | 1 | 111110 | 24 | 101000* | 1 |
| 100010 | 0 | 001100* | 2 | 111111 | 53 | 110001* | 19 |
| 100011 | 0 | 001101* | 1 |  |  | 110101* | 23 |
| 110010 | 6 | 001110* | 1 |  |  | 111001* | 8 |
| 110011 | 6 | 010011* | 1 |  |  | 111101* | 10 |
| 111100 | 9 | 010100* | 1 |  |  |  |  |

"*" indicates a non-ideal mastery mode.

Table 8 Knowledge state, Attribute mastery level and Number of Subjects

| **Knowledge state** | **Attribute mastery level** | **Number of People** | **Knowledge state** | **Attribute mastery level** | **Number of People** |
| --- | --- | --- | --- | --- | --- |
| 000000 | 0 | 0 | 110011 | 4 | 6 |
| 100000 | 1 | 1 | 111100 | 4 | 9 |
| 110000 | 2 | 12 | 111010 | 4 | 8 |
| 111000 | 3 | 10 | 111011 | 5 | 7 |
| 110100 | 3 | 12 | 110110 | 4 | 6 |
| 100010 | 2 | 0 | 110111 | 5 | 7 |
| 100011 | 3 | 0 | 111110 | 5 | 24 |
| 110010 | 3 | 6 | 111111 | 6 | 53 |
